# Effect of Myristica fragrans extract on total body composition in cafeteria diet induced obese rats

**DOI:** 10.6026/97320630015657

**Published:** 2019-10-13

**Authors:** Vangoori Yakaiah, Anusha Dakshinamoorthi, Subramanian Kavimani

**Affiliations:** 1 Santhiram Medical College, Nandyal (AP).(PhD)-SRIHER, Chennai; 2 Sri Ramachandra Institute of Higher Education and Research. Chennai; 3 Mother Theresa Post Graduate and Research Institute of Health Sciences, Pondicherry

**Keywords:** Obesity, BMI, abdominal circumference, total fat, adiposity index, tetra hydrofuran

## Abstract

It is of interest to evaluate the effect of Myristica fragrans on body composition of cafeteria diet induced obese rats. Thirty rats (150-160g) grouped into 5 and each group contains 6 rats. Group-1 was normal control and 2-5 groups were fed with cafeteria diet for 15 weeks to induce obesity. From 16th week to 25th week test drugs were given as mentioned in the experimental protocol. Body weight, BMI, changes in body composition was measured by TOBEC, adipose tissue weights, organ weights, abdominal circumference were measured according to standard methods. After 70days of treatment with MFE 200mg/kg, 400mg/kg Body weight reduced by 9.29%, 12.87% respectively. BMI was also decreased. Abdominal circumference, total fat percentage, organ weights, was substantially reduced. At 400mg/kg of MFE has shown maximum potentiality when compared with 200mg/kg. Orlistat 50mg was used as standard drug. Tetrahydrofuran, flavonoids, saponins, present in Myristica fragrans has shown anti obesity activity. Our findings explain the potentiality of phytochemicals as a potent anti obesity agent, provide scientific evidence for its traditional use and suggest the possible mechanism of action.

## Background

 Obesity is one of the biggest problems in developed and developing countries because of urbanisation and sedentary life style. Its treatment becomes very difficult due to its multiple factors. One of the main causes is high intake fat food and less physical activity [1]. If proper care not taken, it may leads to excessive body growth with BMI more than 30 and related consequences such as cancer, aging, cardiovascular diseases and number of other pathological conditions including type-2 diabetes [2,3]. As per WHO report, nearly 2.8 million people are dying every year because of overweight and obesity related health issues [4]. Globally, it is estimated that over 205 million men and 297 million women were obese, which account for a total of more than 600 million adults worldwide [5]. The rapid increase in the incidence of obesity during only the past few generations is primarily attributed to excessive consumption of palatable energy-dense foods which are high in saturated fats, refined sugars and sodium, combined with sedentary lifestyles [6]. The use of anti obesity drugs to reduce body weight has gained attention in recent years by decreasing the consumption or absorption of food or by increasing energy expenditure [7]. The available anti obesity medications like Sibutramine cause undesirable side effects including heart related complications, hypertension, constipation, psychiatric side effects. Orlistat is another drug approved by FDA for long term management of weight loss. It is a lipase inhibitor to reduce dietary fat absorption by approximately 30% and proved to be useful in both weight loss and weight maintenance. Although Orlistat has approved by FDA, it associates with gastrointestinal complications which include diarrhea, flatulence, bloating, abdominal pain, and dyspepsia. Recently liver injury also has been reported with the use of orlistat [8]. In the last few decades, plant medication has been gaining more importance in the field of herbal research and the usage of plant derived medicine for the treatment of chronic diseases is increasing globally. Herbal medicines for the treatment of obesity and weight reduction have awakened new hope for the invention of new drugs [9]. These herbal medicines became the subject of interest due to its natural origin, cost effectiveness and minimal side effects. Currently, obesity treatment with herbal drugs is the best alternative treatment strategy with fewer side effects and low cost [10]. Myristica fragrans (mace) which is commonly known as Nutmeg belongs to the family Myristicaceae and is an evergreen aromatic tree [11]. Nutmeg probably was imported into Europe during the 12th century by Arab merchants. For many years, this spice has been used as an aromatic stimulant, abortifacient, antiflatulent, and to induce menses [12,13]. Later on it was also used to support digestion and to treat rheumatism. Myristica fragrans seed is also used for decreased appetite, diarrhoea, mouth sore and insomnia. It has many pharmacological actions linked with weight reduction and normal body weight maintenance. Many medicinal effects of Myristica fragrans both in vitro and in vivo has been evaluated, and it has been revealed that Myristica fragrans extract play a preventive role against adipose tissue growth by activating AMPK enzyme in differentiated C2, C12 cells [14]. Previous studies have shown the cholesterol lowering activity of M. fragrans seed extract in rabbits, which contains tetrahydrofuran THF [15], lignans, flavonoids, saponins, and tannins. These chemical compounds are believed to have weight reducing and hypolipidemic properties [16]. Saponins inhibit pancreatic lipase activity. THF regulates the body energy metabolism and prevents the growth of adipose tissue mass [17]. Ethanolic extract also has shown its pancreatic lipase, anti oxidant property [18], glucose and food intake lowering, weight reducing action, and hypolipidemic properties. [19]. According to all these scientific evidence, Myristica fragrans might be useful in the prevention and treatment of obesity by limiting dietary fat digestion, absorption and accumulation in adipose tissue. Animal models have provided major contributions to the investigations of various complex diseases including obesity [20]. Cafeteria (CAF) diets are the closest equivalent to the ultra-processed food diet of humans. This diet type provides animals with free access to 'cafeteria-type foodstuffs' (ultra-processed, energy dense foods) along with laboratory chow ad libitum, so that they have a free choice in what they consume. It provides a robust model of obesity as it mirrors the key obesogenic features of the human diet, maintaining its nutritional and sensorial diversity, and induces similar behavioural and physiological responses associated with human obesity [6]. They are very useful and widely used in obesity research as they readily gain weight and reached obesity in just few months of feeding with cafeteria diet [21,22].Therefore in the present study, the anti-obesity effect of Myristica fragrans was evaluated using swiss albino rats fed a cafeteria fat diet (CD).

## Methodology

### Collection of plant material and preparation of extract:

Fresh Myristica fragrans (Nutmeg) was purchased from wholesale grocery store and it was authenticated by Dr. K.Venkata Ratnam Ph.D. Assistant Professor of Botany, Rayalaseema University. Kurnool, AP. (Herbarium number: 201/2015-16). Dried nutmeg was ground to a fine powder. This powder was used for the preparation of extract with different solvents (petroleum ether, chloroform, and ethanol) by using Soxhlet apparatus for 24 hrs [23]. The yield extract was stored and used for the further analysis and research study.

### Extract selection: 

Among the three solvent (petroleum ether, chloroform, and ethanol) extracts, ethanolic extract contained high levels of polyphenols, saponins, tannins, alkaloids, flavonoids and triterphenoids. Tannins and flavonoid has shown free radical scavenging activity and pancreatic lipase inhibitory effect in animal models of obesity [24,[25]. Based on the potentiality of ethanolic extract, it was chosen for the present study. 

### Dose selection:

The acute oral toxicity study was performed as per Organization for Economic Co-operation and Development (OECD) guidelines. The dose 2000mg/kg showed neither visible sign of toxicity nor mortality till the end of the study period 14 days. The results clearly indicated non-toxicity of the dose of 2000mg/kg. The ethanolic extract tested and it was safe and nontoxic. From the LD50, the experimental doses selected as 1/10th and 1/5th and considered as Test-1, Test-2. i.e., 200 mg/kg, 400 mg/kg respectively to carry out present study.

### Experimental Animals: 

All experiments related to diet induced obesity were carried out with Wister albino rats. Experiments were conducted at Department of Pharmacology, Santhiram Medical College, Nandyal. Reg. No; (897/PO/Re/S/05/ CPCSEA). After initial acclimatization for 7 days, the animals were grouped into five. All procedures were conducted as per Institutional Animal Ethical Committee permission (No: (IAEC/SRMC/2017/2). Thirty healthy albino rats weighing between 150-160 randomly divided into 5 groups (Table 1).

### Induction of obesity:

Normal control rats (Group-I) were fed with standard pellet diet of standard composition containing all the recommended macro and micronutrients prepared according to AIN-93 guidelines with water ad libitum. Group-II to Group-V rats were initially fed with cafeteria diet (CD) for 15 weeks to induce obesity [26] and from 16th week on wards, different doses of Myristica fragrans (200, 400 mg/kg b.wt) were supplemented for 70 days (10weeks) along with CD as mentioned. Total study period was 25 weeks.

### Cafeteria diet (CD)/high fat diet:

It consisted of 3 variants as described elsewhere [27]. (1) Condensed milk (8g) + bread (8g) + peanuts (8g) + pellet chow; (2) Chocolate (3g) + biscuits (6g) + dried coconut (6g) + pellet chow and (3) Cheese (8g) + boiled potatoes (10g) + pellet chow. The different variants were fed on three alternate days throughout the treatment period (15wks).

### Determination of body weight:

Throughout the experimental period the weight gain of rats was monitored .Body weight was measured on day 1 and once in 10 days up to 70 days. Initial weight means of the animals and at the end of 70th day, final weight means was calculated. Mean difference was shown in bar diagrams ([28].

### Body mass index (BMI):

BMI was calculated before and after treatment as an index of obesity. The body weight and body length were used to determine the BMI. Body length (nose-to-anus) was determined in all rats at 70th day of treatment in anaesthetized rats by using the formula. BMI= Body weight (g)/naso-anal length (cm2) [29].

### Abdominal circumference (AC): 

The abdominal circumference i.e., immediately anterior to the forefoot was determined, on the 70th day of the study in all thirty anaesthetized rats. Abdominal circumference (AC) was measured on the largest area of the rat abdomen using a plastic non-extensible measuring tape with an accuracy of 0.1 cm. Rats were placed in ventral position. Mean and percentage of reduction of AC was presented in results [30].

### Body composition by total body electrical conductivity (TOBEC):

**Figure 1:**
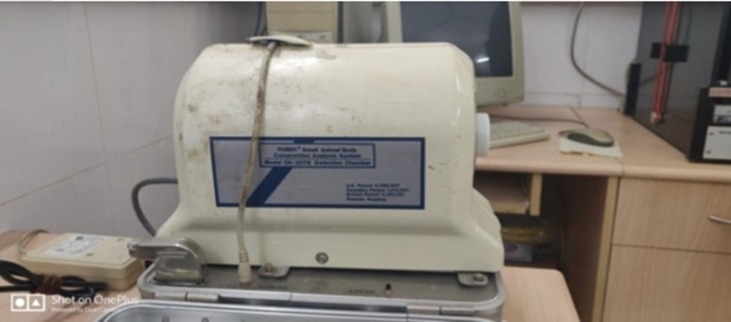
The body composition analysis system (EM-SCAN) is shown

For all experimental groups of rats, body composition was assessed by using scanning machine at the end of study period (on 70th day). It was done by Total Body Electrical Conductivity (TOBEC) using small animal body composition analysis system (EM-SCAN, Model SA-3000 Multi detector, Springfield, USA) as shown in Figure 1. Lean body mass, fat-free mass and total body fat, fat percentages were calculated as per manufacturer's protocol.

The following body composition parameters were obtained mathematically, where E stands for total electrical conductivity.

Total fat = Total body weight-Lean mass

Fat percentage= (Total fat)/(Total body weight) x 100

Lean mass: 0.5 x E+0.3 x total body weight 

Fat free mass: 16.28+0.4 x E

The above prediction equations have been reported to be very accurate for Wistar, Sprague Dawley, F-344 N, CFY, WKY and Holtzman rats [31].

### Organ weights and Adipose tissue (fat pad) weights:

At the end of the experimental period, rats were fasted overnight and anesthetized with isoflurane and sacrificed. Organs such as liver, Heart, kidney, and Adipose tissues (Mesentric, retroperitoneal, epididymal fat pads) from each rat were surgically removed, after detailed necropsy examination, wet weights were measured with experimental electrical balance (Shimadzu) and stored at - 800C for further studies [32].

### Adiposity index:

Adiposity index (AI), a measure of the total weight of the visceral fat depots (epididymal, retroperitoneal and mesenteric) in the body, was determined according to Taylor and Phillips method using the below formula: 

Adiposity Index (AI%) = (Peritoneal WAT+ Mesentric WAT+ subcutaneous WAT/Body weight)*100

Adipose tissue (Epididymal, retroperitoneal and mesenteric fat depots) were isolated, freed from surrounding tissues, weighed individually and after that total weight was calculated.[33].

## Results

### Preparation of plant extract: 

Extract was prepared with nutmeg powder by using Soxhlet apparatus by continuous hot extraction process successively with three different solvents Petroleum ether, chloroform, and Ethanol based on their polarity for 24 hrs.

The percentage of yield extract = (extract obtained/total powder used) x 100 

### Morphological Parameters of Obesity: 

The obesity and body composition was assessed by evaluation of morphological parameters (Body weight, percentage of difference in body weight, BMI, Abdominal circumference, total body scanning, organ and fat pad weight (Epididymal, Mesenteric and Retroperitoneal fats), Adiposity index), was determined. Along with morphological parameters, serum lipid profile was also estimated.

### Body weight:

**Figure 2:**
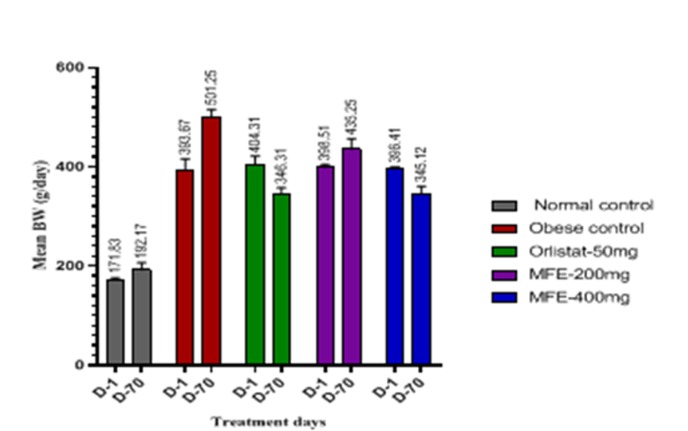
Effect of MFE on Body weight (g/day); The values are represented as Mean±SD (n=6) statistically analysed by one-way ANOVA followed by Post hoc Tukey's test.***p<0.001 extremely significant, **p<0.01 very significant, *p<0.05 significant, ns p>0.05 no significance

**Figure 3:**
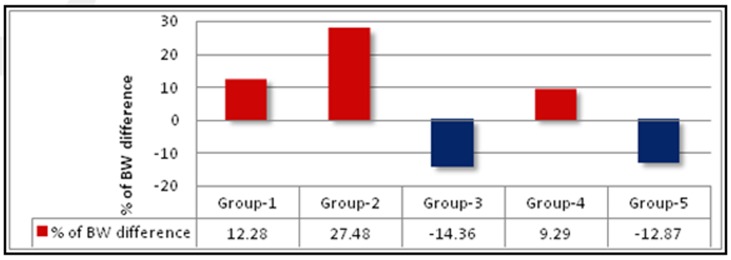
Percentage of body weight difference after 70 days of treatment.*p<0.05, **p<0.01 Vs Normal control; # p<0.05, ## p< 0.01 Vs Obese control

The changes in body weight in different groups of animals during the experiment were showed in Figure 2 and Figure 3. When normal control group of rats (NC) were compared with rats fed on high fat diet (HFD), a substantial gain in body weights observed in CD-fed groups (Table 2). However, Oral supplementation with MFE (400mg) or orlistat significantly (P < 0.001) reduced body weight as compared to the obese control group but there was less action with low dose of MFE (200mg/kg).

### Body mass index (BMI):

**Figure 4:**
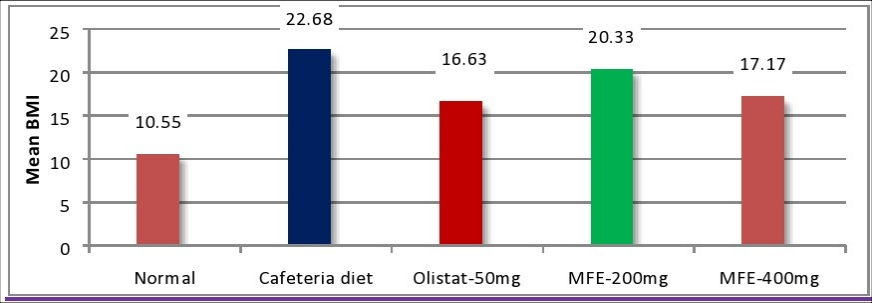
Effect of MFE on BMI on the 70th day. *p<0.05, **p<0.01 Vs Normal control; # p<0.05, ## p< 0.01 Vs Obese control

The mean body mass index was significantly increased in high fat diet fed group (group-II) as compared to the normal control rats (group-I). While body mass index was significantly decreased in high dose of MFE (400 mg/kg-group IV) and Orlistat (50 mg/kg-group III) but with low dose of MFE (200 mg/kg-group-V) less action elicited when compared to obese control group (II) (Figure 4 and Figure 7). According to Rabiu et al. normal BMI: 0.45-0.68 [28].

### Abdominal circumference (AC):

**Figure 5:**
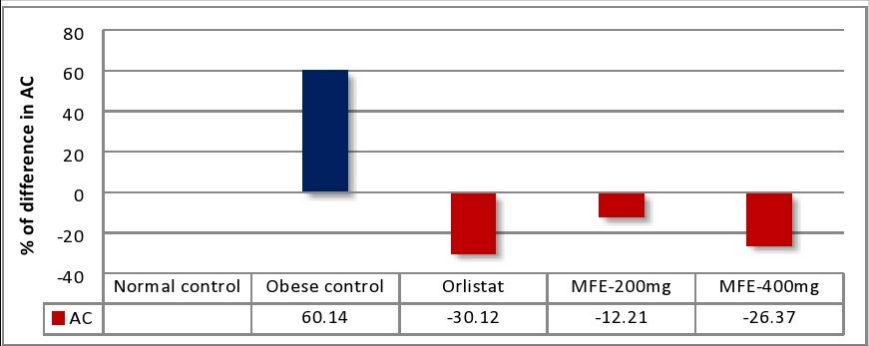
Effect of MFE on abdomina circumference in 70 days of treatment. *p<0.05, **p<0.01 Vs Normal control; # p<0.05, ## p< 0.01 Vs Obese control

In part of the morphological study of obese rats, abdominal circumference was measured for assessing visceral fat mass [34]. Abdominal circumferences were significantly increased by 37.55% in obese control group when compared with normal control group (P<0.01, Figure 8). Whereas, MFE 200mg, 400mg/kg treated group showed significant decrease in abdominal circumferences by 12.21% and 26.37% respectively, after treatment of 70 days (P<0.05). Also, orlistat treated group showed significant reduction in abdominal circumference by 30.12% (P<0.01). These results revealed that MFE has significant effect on AC reduction when compared with obese rats (Table 3 and Figure 5).

### Effect of MFE on body composition in obese rats:

**Figure 6:**
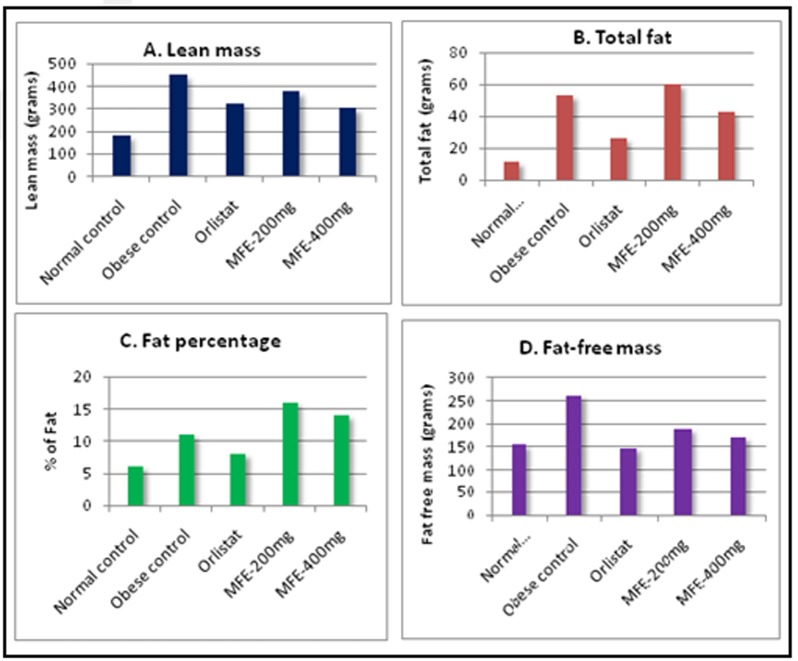
Effect of MFE on body composition (A, B, C, D). *p<0.05, **p<0.01 Vs Normal control; # p<0.05, ## p< 0.01 Vs Obese control

Figure 6 (A, B, C, D) depicts the changes in body weight and body composition of experimental rats. Consumption of cafeteria diet for 15 weeks produced a substantial increase in body weight (501.34±14.62g), total fat (53.6±7.2g), fat % (11.2±4.4), and fat free mass (262.4±3.4g) in obese control group when compared to normal control group of rats whose body weight, total fat, fat % and fat free mass were 192.17±14.67g, 11.4±8.3g, 6.2±4.7% and 155.3±5.2g respectively. Oral administration of MFE (200, 400mg/kg) for 70 days considerably reduced body weight and body composition in a dose dependant manner. Among the two doses administered, MFE at a dose of 400mg/kg, showed significant (p < 0.05) therapeutic effect. At 400mg/kg of MFE, the body weight, total fat, fat % and fat free mass were 345.12±15.67g, 43.3±2.1g, 14.66±4.2% and 172.5±6.5g respectively (Table 4).

### Organ weights and adipose tissue (fat pad) weights:

**Figure 7:**
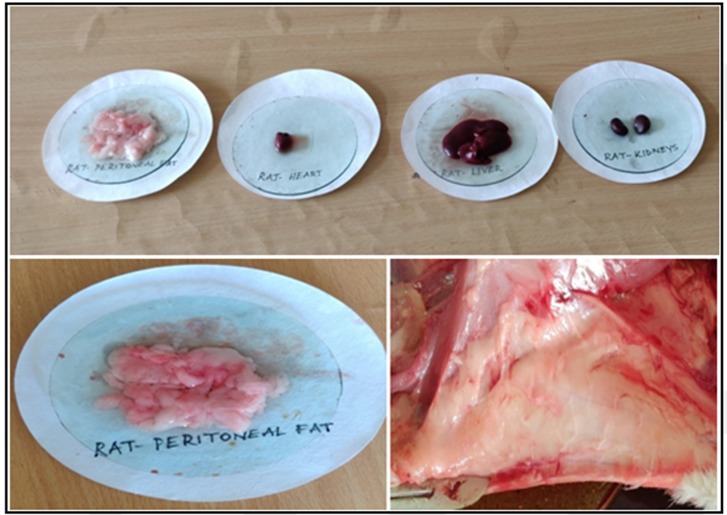
Picture showing separated fat and organs from the rat's body

At the end of the experimental period, animals were anesthetized with isoflurane and sacrificed. Organs such as liver, Heart, kidney, and Adipose tissue (fat pads) from each rat were surgically removed, and wet weights were measured with experimental electrical balance and stored at-800C for further studies. After the end of the experimental period, rats were anesthetized with isoflurane and sacrificed, weighed all organs such as liver, kidney, and heart and Fat pads (Table 5).Weights of organs (Liver, Heart, and kidney) and fat pad weights (Epididymal, Retroperitoneal and Mesenteric) of obese rats when fed with cafeteria diet increased considerably which were significantly (p < 0.05) and dose dependently reduced by MFE treatment (200 and 400mg/kg) after10 weeks. Decreased the weights of liver, heart, and kidney and fat pad weight showed a reduction in Myristica treated groups (Figure 7).

### Adiposity index:

Adiposity index (AI), a measure of the total weight of the visceral fat depots (epididymal, retroperitoneal and mesenteric) in the body, was determined according to Taylor and Phillips method using the below formula:

Adiposity Index (AI) (%) = [Σ (Peritoneal WAT+ Mesentric WAT+ subcutaneous WAT / Body weight X 100]

**Figure 8:**
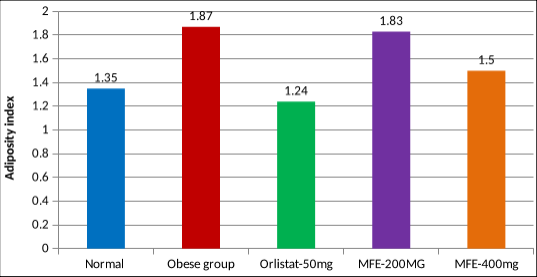
Effect of MFE and Orlistat on adiposity index. *p<0.05, **p<0.01 Vs Normal control. # p<0.05, ## p< 0.01 Vs Obese contro

Adipose tissue (Epididymal, retroperitoneal and mesenteric fat depots) were isolated, freed from surrounding tissues, weighed individually and after that total weight was calculated. Adiposity index of experimental rats feeding on CD (HFD) considerably increased (7.1) the adiposity index. After treatment with Myristica fragrans extract (200, 400 mg/kg) for 70 days decreased the adiposity index (6.3 and 5.6 respectively) but less effective than Orlistat (3.4). The state of AI in different groups showed in Figure 8.

## Discussion

The increased prevalence of obesity across the world has necessitated exploring suitable therapeutic alternatives. Although there is a growing interest for herbal remedies across the globe, lack of adequate systematic studies and scientific evidences on plants and herbs is limiting their usage. Hence, more detailed herbal studies are needed on suitable animal models. Rats were used in the study because their metabolic and physiological systems work similarly as human beings. Ethanolic extract contained high levels of phyto constituents, hence used in the study. Rats were fed with high fat Cafeteria Diet (CD) for 15 wks to induce obesity. In present generation cafeteria diet having major role in obesity, metabolic complications and ovarian dysfunction, reduced folliculo genesis [35,36]. We observed an increase in body weight and fat per cent, which is a hallmark of obesity. Long-term CD feeding resulted in obesity which was linked with increased weight, lean mass, total fat, fat percentage and fat free mass [37]. This might be due to consumption of a diet rich in calories in the form of sugars and fats and its accumulation in various parts of the body, leading to excessive growth of adipose tissue [38]. Oral administration of Myristica fragrans extract significantly reduced body weight, and total body composition (lean mass, total fat, fat percentage and fat free mass) in experimental rats, compared with that of CD control rats. This suggests that MFE may inhibit lipid absorption, transportation and accumulation in adipose tissue by inhibiting pancreatic lipase enzyme. Inhibiting pancreatic lipase would facilitate fat non-digestion and absorption leading to lesser lipid intake. Accumulation of excessive fat in adipocytes is the underlying phenomenon for obesity. MFE administration has effectively lowered the CD-induced elevated levels of lipid profiles [39] suggesting the therapeutic potential of polyphenols and triterpenoids present in Myristica fragrans extract. The presence of phytoconstituents such as flavonoids, Tetrahydrofurans, (which acts on C2, C12 cells) [14], Lignans, and related compounds may play a prominent role in therapeutic activity of MFE. Together, based on morphological, biochemical, and histological analysis we conclude that ethanolic extract of Myristica fragrans has potent anti adipogenic and anti obesity activities. These findings suggest that MFE can be used as a potential therapeutic alternative for the treatment of obesity with no side effects.

## Conclusion

Overweight and obesity are commonly neglected health issues. In recent decades obesity has reached to high levels in both the developed and developing world. The present work was aimed to evaluate the anti-obesity activity of Myristica fragrans. Ethanolic extract of Myristica fragrans showed more phytochemicals. So it was selected. For studying anti-obesity activities, rats were fed with high fat diet (cafeteria diet)-CD, and the MFE was administered. CD has substantially altered morphological and biochemical aspects. Administration of MFE reduced significantly (p < 0.05), body weight, total body composition (lean mass, total fat, fat percentage and fat free mass), BMI, abdominal circumference (AC), total fat pad percentage, lipid profile, Atherogenic index (AI), organ weights and adiposity index (AI) in CD-fed groups in a dose dependent manner (200,400 mg/kg b.wt). With all these parameter alterations (increased in obese models and decreased in MFE treatment models) we came to conclusion that Myristica fragrans extract can alter the body composition. The presence of phyto constituents such as tetra hydro furans, lignans, saponins, tannins, flavonoid, and poly phenols in high percentage in ethanolic extract may play a prominent role in therapeutic activity in obesity. The probable mechanism might be pancreatic lipase inhibition, activation of AMP-Kinase in C2, C12 cells, and hunger sensory mechanism inhibition as per previous scientific reports [14,15] and [18, 19].This study demonstrates the anti hyper lipidemic and anti-obesity potential of Myristica fragrans extract and offers scientific validation and basis to develop anti-obesity drugs.

## Figures and Tables

**Table 1:** Research design and grouping of animals

S. No	Group	Treatment	No. of animals
Group-1	Normal control	Standard pellet diet	6
Group-2	Obese control	Cafeteria diet-CD	6
Group-3	Standard drug	CD+Orlistat-50mg/kg	6
Group-4	Test-1 control	CD+MFE-200mg/kg	6
Group-5	Test-2 control	CD+MFE-400mg/kg	6

**Table 2:** Myristica fragrans extract yielded with different solvents

Solvent	Mace powder weight	Extract obtained	Percentage of extract yield(w/w)
Ethanol-300ml	30 g	5g	16.60%
Petroleum ether-300ml	30 g	4.5g	15%
Chloroform-300ml	30 g	4.8g	16%

**Table 3:** Effect of Myristica fragrans extract on abdominal circumference on 70th day

		% of reduction in AC
Rats	Mean AC (cm)	(Treatment Vs Obese control)*
		(Obese Vs Normal)#
Group-1 (NC)-SPD	9.66±1.26	-
Group-2 (Obese)-CD	15.47±1.31	+60.14#
Group-3(Orlistat-50)	10.81±1.13	-30.12*
Group-4(MFE-200mg)	13.58±1.15	-12.21*
Group-5(MFE-400mg)	11.39±1.32	-26.37*
The values are represented as Mean±SD (n=6) statistically analysed by one-way ANOVA followed by Post Hoc Tests. ***p<0.001 extremely significant **p<0.01 very significant, *p<0.05 significant, ns p>0.05 no significance

**Table 4:** Effect of Myristica fragrans on Total body composition in obese rats

Physical parameters	Body weight (g)	Lean mass (g)	Total fat (g)	Fat %	Fat free mass (g)
Group-1 (NC)-SPD	192.17±14.67	181±16.52	11.1±8.3	6.2±4.7	155.3±5.2
Group-2 (Obese)	501.34±14.62**	448±32.12**	53.6±7.2**	11.2±4.4**	262.4±3.4***
Group-3 (Orlistat50mg)	346.31±11.23##	320±41.35##	26.2±6.4##	8.9±2.8##	148.2±5.6##
Group-4(MFE-200mg)	435.25±21.51#	375±11.25#	60.1±5.4#	16.4±6.2#	190.7±2.1#
Group-5(MFE-400mg)	345.12±15.67##	302±16.21#	43.3±2.1##	14.66±4.2##	172.5±6.5##
The values are represented as Mean±SD (n=6) statistically analysed by one-way ANOVA followed by Post Hoc Tests. ***p<0.001 extremely significant **p<0.01 very significant, *p<0.05 significant, ns p>0.05 no significance.

**Table 5:** Effect of MFE on body organs and fat pads weight

GROUPS	Liver(g)	Kidney(g)	Heart(g)	Peritoneal (g/100bw fat)	Mesentric g/100bw fat)	Subcutaneous (g/100bw fat)
Group-1 - Normal Control	7.4±0.2	2.3±0.1	0.9±0.1	0.8±0.2	0.7±0.2	1.1±0.4
Group-2 - Obese Control	12.3±0.4*	3.6±0.4**	1.7±0.4*	3.8±0.4*	2.2±0.4**	3.4±0.4*
Group-3 - (Orlistat 50mg/kg	9.2±0.5##	2.1±0.4##	1.0±0.2	1.6±0.2##	1.3±0.2#	1.4±0.1##
Group-4 CD+MEF 200mg/kg	11.6±0.4	3.2±0.7#	1.5±0.6	3.1±0.8ns	2.1±0.3ns	2.8±0.5
Group-5 - CD+MEF 400mg/kg	10.4±0.1#	2.6±0.1#	1.2±0.7#	2.1±0.1#	1.2±0.2#	1.9±0.4#
The values are represented as Mean±SD (n=6) statistically analysed by one-way ANOVA followed by Post Hoc Tests. **p<0.001 extremely significant **p<0.01 very significant, *p<0.05 significant, ns p>0.05 no significance
